# Towards Efficient Implementation of an Octree for a Large 3D Point Cloud

**DOI:** 10.3390/s18124398

**Published:** 2018-12-12

**Authors:** Soohee Han

**Affiliations:** Department of Geoinformatics Engineering, Kyungil University, Gyeongsan 38428, Korea; scivile@kiu.kr

**Keywords:** octree, 3D point cloud, terrestrial laser scanning, memory-based octree, file-based octree

## Abstract

The present study introduces an efficient algorithm to construct a file-based octree for a large 3D point cloud. However, the algorithm was very slow compared with a memory-based approach, and got even worse when using a 3D point cloud scanned in longish objects like tunnels and corridors. The defects were addressed by implementing a semi-isometric octree group. The approach implements several semi-isometric octrees in a group, which tightly covers the 3D point cloud, though each octree along with its leaf node still maintains an isometric shape. The proposed approach was tested using three 3D point clouds captured in a long tunnel and a short tunnel by a terrestrial laser scanner, and in an urban area by an airborne laser scanner. The experimental results showed that the performance of the semi-isometric approach was not worse than a memory-based approach, and quite a lot better than a file-based one. Thus, it was proven that the proposed semi-isometric approach achieves a good balance between query performance and memory efficiency. In conclusion, if given enough main memory and using a moderately sized 3D point cloud, a memory-based approach is preferable. When the 3D point cloud is larger than the main memory, a file-based approach seems to be the inevitable choice, however, the semi-isometric approach is the better option.

## 1. Introduction

Advances in 3D terrestrial laser scanning technology and its various applications have increased the size of 3D point clouds enormously. Unlike elements stored in conventional spatial database management systems (SDBMS), a 3D point cloud has even more entities—points, up to billions in number, however, each entity is not topologically related to the others. Thus, it is necessary to use relevant methods to handle the data. The methods can be categorized into two: lossy compression or abbreviation, and lossless indexing. The former category eliminates less meaningful points from the 3D point cloud. Several relevant approaches have reported that the reduced data still exhibits consistent results with half or even less point density [1,2,3]. The latter category retains and uses the original coordinate information of all points, and then uses special data structures to store and retrieve the data efficiently. For example, as a dynamic partitioning algorithm, R-tree is commonly utilized in SDBMS with its derivatives, and might be applicable for this purpose. However, R-tree is based on minimum bounding rectangles (MBR) and points are apt to be enclosed by overlapping nodes, making it a poor solution [4]. K-d tree, which is also a dynamic partitioning algorithm is more efficient and has been officially implemented in the point cloud library (PCL) [5]. However, in the worst case, all of the child nodes should be retrieved to traverse from a node to its child node where the 3D boundary satisfies a positional query [6]. Thus, a large 3D point cloud necessitates proper methods to re-organize or index itself efficiently. Among the known methods, octree is popular for its memory efficiency, query speed and structural simplicity [7]. In octree, only one child node in each depth is traversed because the 3D boundary of each node is implicitly known by positional query. Thus, a leaf node can be advantageously retrieved in this approach. Octree is now being exploited by a number of applications for segmentation and visualization of 3D point clouds [8,9,10,11,12,13,14], and also in PCL. However, octree, as a static partitioning algorithm, has a potential weakness, that is, memory waste because eight child nodes are always declared, even when not all of them bear point(s) within themselves. To address this weakness, a schema was presented to avoid declaration of child nodes that bear no points, and to terminate subdivision if the number of points goes below a threshold after further subdivision [15,16]. A memory efficient encoding method was also employed to minimize the size of a node in octree.

The present study uses a native octree, which means that derivations of octree, as introduced in the relevant approaches [15,16] are not considered. Instead, it deals with other issues that influence the performance of octree. These include how to make a compact node in a native octree, how to design functions (methods) applicable to the node, where to store and retrieve the 3D point cloud itself (main memory or HDD), and what the shape of octree should be. From preliminary tests, several influencing factors were found: 1) the size of a node is very flexible but does not have much influence on query speed, and 2) dynamic declaration of nodes in the octree construction process claims more memory than expected, thus, array declaration is preferable after a pseudo octree construction [17]. The present study is based on the array-based approach and this will not be further mentioned in this paper. The paper is structured as follows: implementation of a compact node and relevant methods are outlined in Section 2.1.1, implementation of a file-based octree to reduce main memory usage is described in Section 2.1.2, more efforts to enhance the performance of octree are discussed in Section 2.1.3 and Section 2.1.4, and its application is shown in Section 2.2. Results along with the discussion and conclusions follow in Section 3 and Section 4.

## 2. Material and Methods

### 2.1. Algorithm Development

#### 2.1.1. Implementation of Octree for a 3D Point Cloud

An octree is defined as a tree data structure in which each internal node has exactly eight children, where a three dimensional space is created by recursively subdividing it into eight octants [18]. To index a 3D point cloud using octree the 3D boundary is divided into eight octants, which are further subdivided recursively only when they bear point(s) within themselves until the sequence reaches a given threshold value, namely depth. The final subdivision results in eight leaf nodes that store points within their archives. In the present study, the basic steps used to implement octree from a 3D point cloud were:

1. An axially-aligned minimum bounding hexahedron (hereafter, MBH) is defined to tightly enclose the whole 3D point cloud and assigned to a head node.
2. Eight new MBHs are defined by halving the MBH along the *x*-, *y*- and *z*-axes, and are assigned to eight child nodes.
3. A child node, of which MBH encloses an input point, is chosen and the input point is passed over a child node in further depth.
4. Step 2 and Step 3 are continued until the depth reaches a given threshold value (hereafter, Depth) and the final child node (hereafter, the leaf node) stores the input point.
5. Every point in the 3D point cloud is assigned to the head node and undergoes Step 2 to Step 4.

A larger Depth creates more subdivision and allows leaf nodes to have fewer points, and a smaller Depth has the opposite effect. Having fewer points in a leaf node reduces computational overhead in point retrieval, but also increases the traversing route from head to leaf node. Thus, Depth should be experimentally adjusted to minimize the overall point retrieval time.

**Figure 1:**
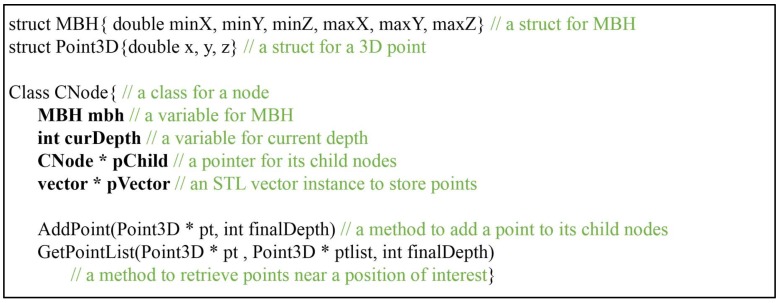
Pseudo codes of a basic form CNode.

Pseudo codes to implement the steps in C++ language based on the standard template library (STL) are given in Figure 1. The Addpoint method selects a child node of which octant encloses an input point, and updates the MBH of the selected child node. The input point is recursively passed over selected child nodes until the depth of the current node (curDepth) equals the final depth (finalDepth). The final selected leaf node pushes back the pointer (pt) of the input point to a vector archive (pVector). The GetPointList method recursively retrieves child nodes enclosing the position of interest by a similar mechanism, and points can be retrieved from the pVector of the selected leaf node. The size of the node class in Figure 1 is determined by the type of variable used in the MBH. The size of CNode is 60 bytes (= mbh(6 × 8 bytes) + curDepth(4 bytes) + pVector(4 bytes) + pChild(4 bytes)) using double precision or 36 bytes (=mbh(6 × 4 bytes) + depth(4 bytes) + pVector(4 bytes) + pChild(4 bytes)) using single precision. In both cases, a great deal of memory is required during tree construction—estimated to be up to $60\times 8^{n}$ or $36\times 8^{n}$ bytes after n-subdivisions, in the worst case.

**Figure 2:**
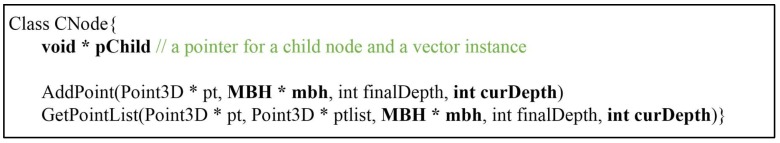
Pseudo codes of a compact form CNode.

**Figure 3:**
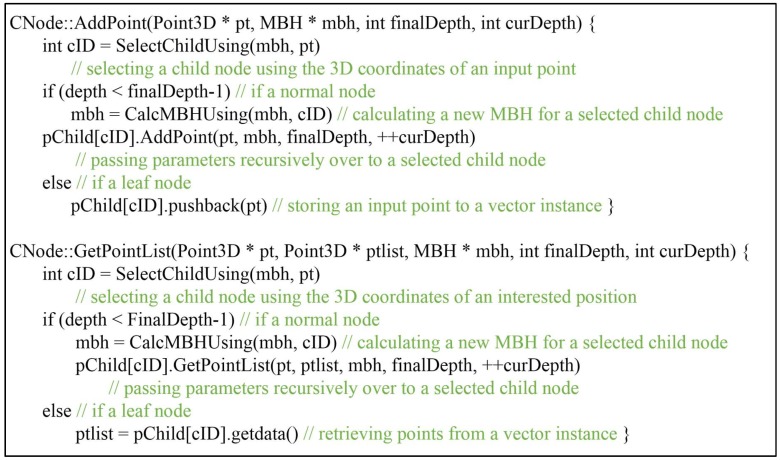
Pseudo codes of the AddPoint and GetPointList methods.

To reduce the size of a node class, most of the variables are omitted and the methods are revised to pass the necessary parameters over to the child nodes. A compact form of a node class declares only a variable pChild, as shown in Figure 2. A void pointer pChild can designate both a child node in a normal node and a vector instance in a leaf node. The AddPoint and GetPointList methods are revised accordingly to pass over more parameters, as shown in Figure 3. AddPoint selects a child node by using the 3D coordinates of an input point and calculates a new MBH for a selected child node in further depth. The new MBH, along with Depth and the input point, are passed over to a child node recursively until a leaf node is reached in which an input point is stored. Likewise, GetPointList operates using a similar mechanism, however it selects a child node not by an input point but by a position of interest and passes an additional parameter (ptlist) over to obtain the queried results. Either way, the size of a node is reduced to the size of a pointer which occupies 8 bytes in 64-bit system.

#### 2.1.2. Implementation of File-Based Octree

**Figure 4:**
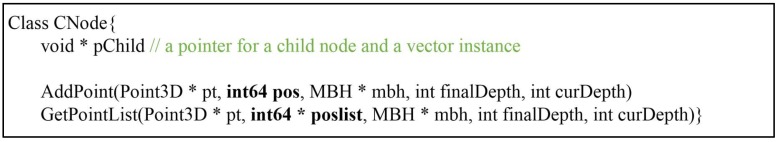
Pseudo codes of a revised form CNode using a file-pointer.

**Figure 5:**
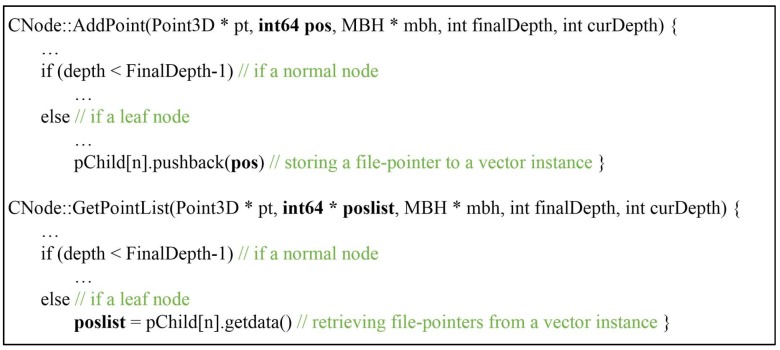
Pseudo codes of the revised AddPoint and GetPointList methods.

The performance of an octree is strongly influenced by the media where the 3D point cloud practically exists. For the best performance, the 3D point cloud should be loaded into the main memory and stored in an array of Point3D struct, as in Figure 1. A pointer to the struct, instead of the 3D coordinates themselves should be put into the head node and passed over to the child nodes until it can be stored in a leaf node. However, the 3D point cloud itself requires a great deal of memory—sometimes more than the main memory. To avoid defects, octree can be constructed based on file-pointers which directly refer to 3D points stored in a hard disk drive (HDD) or a solid-state drive (SSD). The AddPoint method is revised to pass over a new file-pointer (pos) which refers to the address of an input point in an HDD or a SSD and is finally stored to a leaf node, as shown in Figure 4 and Figure 5. GetPointList is also revised to substitute a new parameter (poslist) for an old one (ptlist) to obtain the queried results in the file-pointer format.

The former method, hereafter referred to as the memory-based method, is enormously faster than the latter, hereafter referred to as the file-based method, during octree-construction and point-retrieval. The latter can save the main memory by omitting loading of the 3D point cloud to the main memory, thus, it is applicable when the size of the 3D point cloud is larger than the main memory. However, the average time to retrieve arbitrary data using a file-pointer in an HDD is 100 times slower than using a normal pointer in the main memory. This means that the file-based method may suffer from slow octree-construction and point-retrieval speed. Nevertheless, the file-based approach is preferable because it can expand the volume of the 3D point cloud to be indexed in an octree.

#### 2.1.3. Implementation of an Anisometric Octree

**Figure 6:**
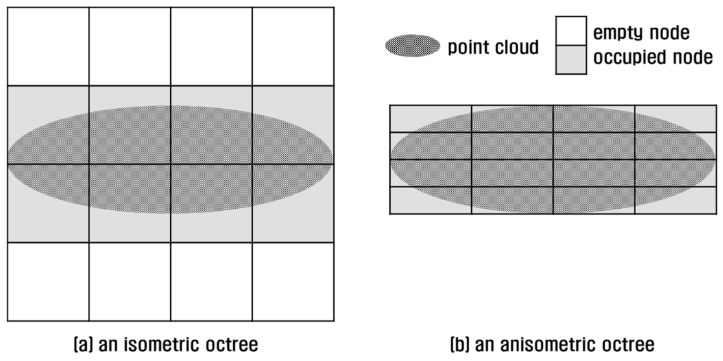
Comparison of octrees: (**a**) an isometric octree; (**b**) an anisometric octree.

An octree is commonly implemented in an isometric shape; that is, the MBH of an octree is cubic-shaped regardless of the original shape of the 3D point cloud. If the 3D point cloud is severely imbalanced in the *x*-, *y*- and *z*-axes, it is preferable to tightly fit the MBH to the point cloud. As seen in Figure 6a, an isometric implementation of octree to cover an oval-shaped 3D point cloud can yield empty nodes. Point concentration in fewer nodes results in load-unbalance and query performance degradation. To avoid this defect, an octree can be implemented in an anisometric shape in which the points are better distributed to more nodes (Figure 6b).

**Figure 7:**
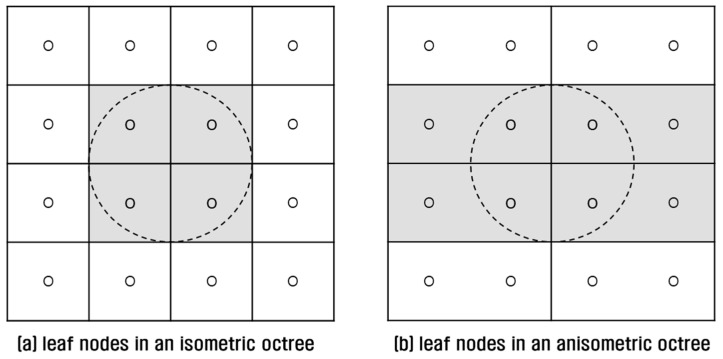
Comparison of leaf nodes: (**a**) in an isometric octree; (**b**) in an anisometric octree.

However, even an anisometric octree can have a negative effect on point retrieval performance. This is because a leaf node inherits the shape of an octree, and an anisometric leaf can increase query overheads. For example, if we retrieve points within a distance from a position of interest in an isometric octree, four leaves are queried and four points are examined (Figure 7a). In an anisometric octree, four leaves are also queried but eight points are examined, causing double query overhead (Figure 7b). As noted earlier, point query is very slow in a file-based octree, and query increment results in severe performance degradation.

#### 2.1.4. Implementation of a Semi-Isometric Octree Group

**Figure 8:**
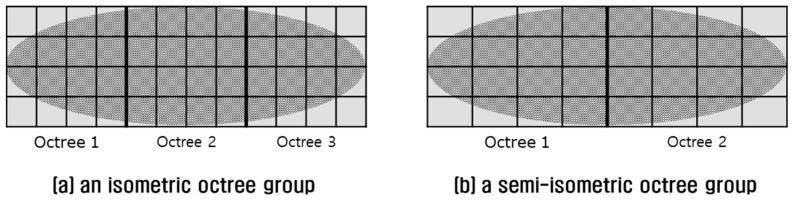
Comparison of octree groups: (**a**) an isometric octree group; (**b**) a semi-isometric octree group.

The tradeoff between isometric and anisometric octrees can be complemented by implementing an isometric octree group. An isometric octree group is composed of isometric octrees which cover the 3D point cloud tightly, but each octree along with its leaf node still maintains an isometric shape. For example, the octree group in Figure 8a resembles the anisometric octree shown in Figure 6b, but its leaf nodes resemble those of the isometric octree in Figure 7a.

An isometric octree group is preferable to achieve better performance in point retrieval. In most cases, however, a perfect isometric octree group is not possible because the length of the larger axis of the MBH is not always an integer multiplication of minor one. Even if possible, an isometric octree group such as in Figure 8a can require three times more memory than a single octree, as in Figure 6b. As an alternative, a semi-isometric octree group is introduced in Figure 8b. It occupies less memory than an isometric octree group and performs better than a single anisometric octree. The shape of an octree in a semi-isometric octree group can be adjusted by controlling a threshold $t_{i}$ which is a number not smaller than one (Equation (1)).

(1) $$l_{s}=\mathrm{argmin}\left(l_{x},\;l_{y},\;l_{z}\right),\;n_{x}=\mathrm{floor}\left(\frac{l_{x}}{t_{i}\times l_{s}}\right),\;n_{y}=\mathrm{floor}\left(\frac{l_{y}}{t_{i}\times l_{s}}\right),\;n_{z}=\mathrm{floor}\left(\frac{l_{z}}{t_{i}\times l_{s}}\right)\;d_{x}=\frac{l_{x}}{n_{x}},\;d_{y}=\frac{l_{y}}{n_{y}},\;d{\;}_{z}=\frac{l_{z}}{n_{z}}$$

where argmin() gives the minimum value among inputs, $l_{x},\;l_{y},\;l_{z}$ denote the lengths of the MBH of the 3D point cloud, $n_{x},n_{y},\;n_{z}$ give the numbers of octrees in the octree group, floor() denotes the largest integer number not larger than an input value, and $d_{x},d_{y},\;d_{z}$ give the lengths of the MBH of an octree. One of $d_{x},d_{y},\;d_{z}$ is equal to $l_{s}$ and the others cannot be larger than $l_{s}\times t_{i}$ by two times. When $t_{i}=1$, for example, it means that any axial length of a single octree is not two times larger than the others and the shape of a single octree is most similar to an isometric one. Given the state of the computational resources, a user can put more weight on memory efficiency or point retrieval performance by adjusting the threshold.

### 2.2. Application to Real Point Clouds

**Figure 9:**
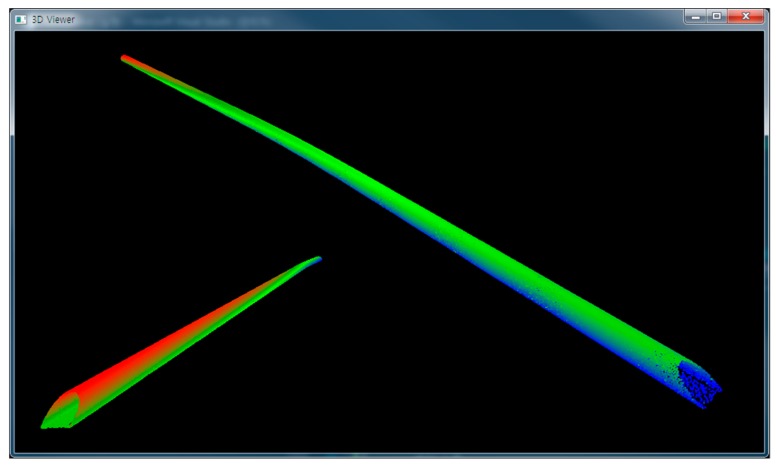
3D point cloud of a long tunnel.

**Figure 10:**
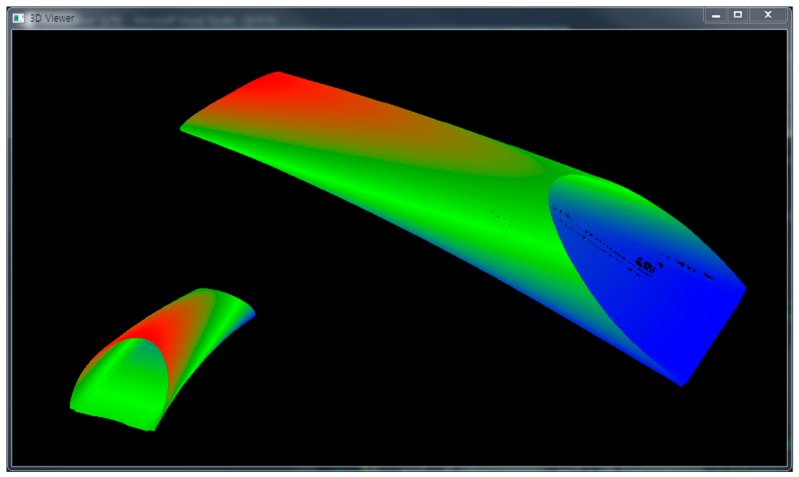
3D point cloud of a short tunnel.

**Figure 11:**
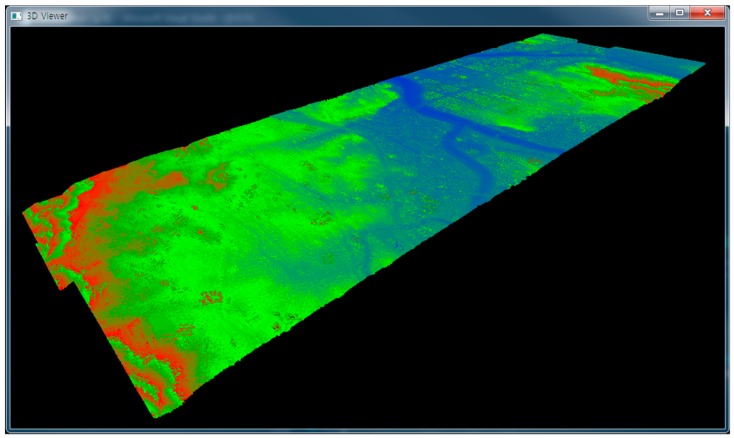
3D point cloud of an urban area.

The three approaches—memory-based octree, file-based octree, and semi-isometric octree groups—were implemented using three 3D point clouds captured in a long tunnel (Figure 9), a short tunnel (Figure 10), and an urban area (Figure 11), respectively. The first and second 3D point clouds were captured by terrestrial laser scanners, and the third by an airborne laser scanner. The first 3D point cloud was composed of 300.5 million points and occupied 6878 MB, the second had 18.4 million points and 420 MB, and the third had 267.5 million points and 6122 MB. The computing system was composed of a 64-GB main memory and a 512-GB SSD. More detailed specifications are shown in Table 1 and Table 2.

## 3. Results and Discussion

As the long tunnel (Data 1) is 1.5 km long horizontally and only 19 m long vertically, the lengths of the MBH are seriously unequal in the *x*-, *y*- and z-directions (Table 1). Memory-based and file-based octrees were constructed in single octrees. The lengths in the x- and y-directions of a leaf node were 29.87 and 69.97 times larger than in the z-direction (Table 3). A semi-isometric octree group was implemented using three thresholds. The group was composed of 171 (= 9 × 19 × 1, threshold = 3) to 1711 (= 29 × 59 × 1, threshold = 1) octrees, where the ratio of the *x*- to *z*-direction ranged from 3.32 (threshold = 3) to 1.03 (threshold = 1) (Table 3). On the contrary, the lengths of the MBH of the short tunnel (Data 2) were 56 m and 26 m horizontally and 12 m vertically, which are not seriously unequal (Table 1). The length in the *x*- and *y*-directions of a leaf node were only 4.68 and 2.13 times larger, respectively, than the length in the z-direction (Table 4). A semi-isometric octree group was implemented using three thresholds and the group was composed of 1 (= 1 × 2 × 1, threshold = 3) to 8 (= 4 × 2 × 1, threshold = 1) octrees (Table 4). The lengths of the MBH of the urban area (Data 3) are 10.7 km and 3.4 km horizontally and 0.3 km vertically, which are very unequal (Table 1). The lengths in the x- and y-directions of a leaf node were 36.89 and 11.65 times larger than the length in the z-direction (Table 5). A semi-isometric octree group was implemented using three thresholds and the group was composed of 36 (= 12 × 3 × 1, threshold = 3) to 396 (= 36 × 11 × 1, threshold = 1) octrees (Table 5).

**Figure 12:**
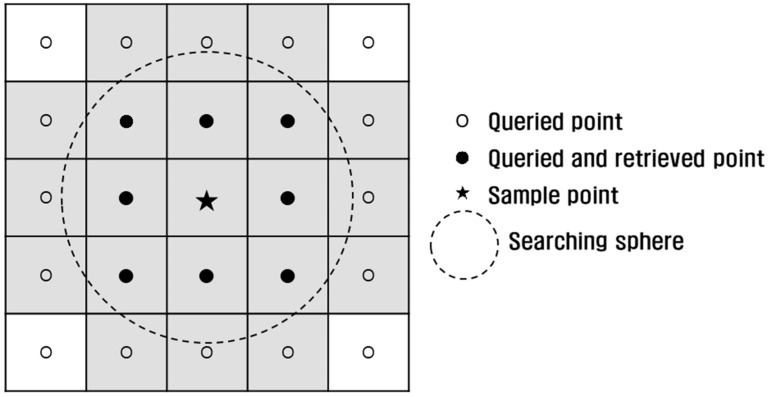
Proximity operation.

The main memory occupancy and time duration were measured during octree construction. To evaluate the performance, a proximity operation was conducted as introduced in [6]. This operation aims to query and retrieve neighboring points within a searching sphere from the sample points (Figure 12). Such an operation is known as fixed distance neighbors (FDN) [19] and can be applied to k-NN [20] if supplemented by distance sorting. The operation is necessary in normal estimation and noise filtering [19,21]. A total of 3005 sample points, or 1/100,000 of the data, were selected from Data 1 and neighboring points within a 5 cm (radius of the searching sphere) were queried. In all methods, the same 1,735,755 points were retrieved, and thus, no faults were detected in the proximity operation. Likewise, the same operation was conducted using Data 2 and Data 3 (Table 6).

Octrees were constructed in Depth 8 to 13 for the memory-based approach and in Depth 8 to 9~11 for the file-based and semi-isometric approaches to avoid memory occupancy exceeding any of the memory-based approach (Table 7, Table 8 and Table 9). Memory usage, along with construction time increased accordingly. As is the precondition, main memory occupancy includes the size of the 3D point cloud itself in the memory-based approach (for example, 6878 MB for Data 1). The memory-based approach exhibited enormous speed in the proximity operation. The result is credible because the performance of the main memory can never be exceeded by a file-based operation, even using SSD. Nevertheless, the semi-isometric approach using Data 1 resulted in a performance that was a little better than the memory-based approach, and quite a lot better than the file-based one. The semi-isometric approach in Depth 8 was defeated once by a file-based one in Depth 13, but the main memory occupancy was almost half. A little more memory occupancy quickly enabled enough performance improvement in the semi-isometric approach in Depth 9. Similar results were observed using Data 2 and Data 3. However, the semi-isometric approach using Data 2 did not result in dramatically better performance than the file-based one because the lengths of the MBH are not seriously unequal in the *x*-, *y*- and *z*-directions.

The best performance for the semi-isometric approach using Data 1 was achieved in Depth 10 with threshold = 1 (Table 10). In this case, the main memory occupancy was 72.76% that of the memory-based approach in Depth 8, but performance increased to 81.82%. In the grey-highlighted cases, the semi-isometric approach achieved better performance than the file-based approach in the same Depth. In the green-highlighted cases, the semi-isometric approach achieved better performance than the best of the file-based approach with less memory occupancy. In the yellow-highlighted cases, the semi-isometric approach resulted in even better performance. The results in the cases of threshold = 3 using Data 3 were almost the same as the file-based approach (Table 8 and Table 11). This can be easily understood by the fact that the two approaches share the same leaf node dimensions (Table 4). Nevertheless, it is clear that the performance of the semi-isometric approach is better than the file-based one in the same Depth in all cases. Thus, it can be said that the semi-isometric approach is a good alternative compared to the other approaches.

Theoretically, a better performance of the semi-isometric approach should be achieved with a smaller threshold $t_{i}$ and a larger Depth. This is because query candidate points are more delicately selected if a leaf node gets more cubic-shaped and smaller. Accordingly, all results using Data 1 meet the expectation (Table 10). However, the best performance was achieved in Depth 9 with threshold = 1 using Data 2, and in Depth 8 with threshold = 2 using Data 3 (Table 11 and Table 12). This is because of over-subdivision of the octree, in which a route to reach a leaf node is so long that it overwhelms the effect of the more delicate selection of query candidate points. For this reason, an optimal parameter is hard to determine before an experiment, and thus, several configurations should be investigated to find the best one.

## 4. Conclusions

In the present study, a basic algorithm to construct an octree for a 3D point cloud is introduced. The algorithm can be improved in terms of memory efficiency by using a compact form of node and revised parameter passing methods, and even further by using a file-based approach. However, the query speed of a file-based approach is very poor and becomes even worse when dealing with very longish 3D point clouds scanned in tunnels and corridors. The defects can be somewhat addressed by avoiding point concentration on fewer nodes using an anisometric approach, but this also brings about the problem of query overhead increment. Finally, the semi-isometric approach was introduced to improve query performance by implementing several semi-isometric octrees in a group. In the experiments, query performance and memory efficiency could be significantly improved in the case of a 3D point cloud captured in a long tunnel. When applied on a 3D point cloud captured in a short tunnel, the semi-isometric approach resulted in better performance (though not dramatically improved) than the file-based approach. Airborne laser scanning data was also tested and the semi-isometric approach resulted in acceptable enhancement of performance. By using media such as HDD of SDD, known to be much slower than main memory, a file-based approach and its derivations can never exceed the performance of a memory-based approach. Therefore, given enough main memory and using a moderately sized 3D point cloud, the memory-based approach is the best choice. When a 3D point cloud is larger than the main memory, as is quite common today, a file-based approach is the inevitable choice. In this case, however, the semi-isometric approach is a better choice no matter whether the 3D point cloud is longish or not. 

In all of the above approaches, however, every insertion of a point to a leaf node increases the main memory usage because a pointer to the point is pushed back to a vector archive of the leaf node. Eventually, the maximum number of points is limited to the size of the main memory. In future work, a more advanced approach is being planned to address this limitation.

## Figures and Tables

**Table 1:** Specifications of data.

	Data 1	Data 2	Data 3
Laser scanner	C10, Leica Geosystems	Scan station 2, Leica Geosystems	ALTM 3070, Optech
Scanned object	A long tunnel	A short tunnel	An urban area
Dimension	Δx=569.16 m Δy=1442.58 m Δz=19.05 m	Δx=56.05 m Δy=25.57 m Δz=11.98 m	Δx=10708.77 m Δy=3380.64 m Δz=290.26 m
Number of points	300,525,406	18,376,726	267,490,366
Data file size (in double precision float)	6878 MB	420 MB	6122 MB

**Table 2:** Specifications of the computing environments.

Item	Description
CPU	Intel Core i7-6700K @ 4.00 GHz
RAM	64.0 GB DDR4
SSD	512 GB
OS	Windows 7 64 bit
Coding language	C++, compiled in 64-bit release mode in Visual studio 2017

**Table 3:** Comparison of leaf node dimensions of Data 1.

	Memory- and File-Based Octree			Semi-Isometric Octree Group					
$t_{i}$	Ratios			Ratios			No. of octrees		
	***x***	***y***	***z***	***x***	***y***	***z***	***x***	***y***	***z***
1	29.87	59.97	1.00	1.03	1.02	1.00	29	59	1
2				2.13	2.07	1.00	14	29	1
3				3.32	3.16	1.00	9	19	1

**Table 4:** Comparison of leaf node dimensions of Data 2.

	Memory- and File-Based Octree			Semi-Isometric Octree Group					
$t_{i}$	Ratios			Ratios			No. of octrees		
	***x***	***y***	***z***	***x***	***y***	***z***	***x***	***y***	***z***
1	4.68	2.13	1.00	1.10	1.07	1.00	4	2	1
2				2.34	2.13	1.00	2	1	1
3				4.68	2.13	1.00	1	1	1

**Table 5:** Comparison of leaf node dimensions of Data 3.

	Memory- and File-Based Octree			Semi-Isometric Octree Group					
$t_{i}$	Ratios			Ratios			No. of octrees		
	***x***	***y***	***z***	***x***	***y***	***z***	***x***	***y***	***z***
1	36.89	11.65	1.00	1.02	1.06	1.00	36	11	1
2				2.05	2.33	1.00	18	5	1
3				3.07	3.88	1.00	12	3	1

**Table 6:** Specifications of proximity operation.

	Data 1	Data 2	Data 3
Number of sample points (ratio to the whole data)	3005 (1/100,000)	3063 (1/6,000)	2675 (1/100,000)
Number of retrieved points	1,735,755	1,319,435	1,528,718
Radius of searching sphere	5 cm	5 cm	5 m

**Table 7:** Performance comparison among octree implementing methods using Data 1.

	Memory-Based Octree			File-Based Octree			Semi-Isometric Octree Group $\left(t_{i}=2\right)$		
**Depth**	Memory usage (MB)	Construction time (s)	Proximity operation time (s)	Memory usage (MB)	Construction time (s)	Proximity operation time (s)	Memory usage. (MB)	Construction time (s)	Proximity operation time (s)
8	9500	49.30	1.62	2607	51.01	290.32	3025	58.41	9.47
9	9617	55.19	0.76	2725	57.03	127.02	3477	66.80	4.24
10	9765	61.53	0.31	2874	63.40	48.63	4784	78.56	2.45
11	10065	68.92	0.19	3174	70.72	22.34	8240	99.67	2.14
12	10868	78.23	0.16	3978	80.03	11.25			
13	12968	91.26	0.17	6077	92.81	5.71			

**Table 8:** Performance comparison among octree implementing methods using Data 2.

	Memory-Based Octree			File-Based Octree			Semi-Isometric Octree Group $\left(t_{i}=2\right)$		
**Depth**	Memory usage (MB)	Construction time (s)	Proximity operation time (s)	Memory usage (MB)	Construction time (s)	Proximity operation time (s)	Memory usage. (MB)	Construction time (s)	Proximity operation time (s)
8	606	3.12	0.05	185	3.26	7.66	187	3.68	5.54
9	625	3.65	0.05	204	3.79	4.07	223	4.29	2.65
10	713	4.37	0.09	293	4.51	2.59	364	5.15	2.20
11	997	5.43	0.30	576	5.52	2.59			
12	1593	7.21	1.44						
13	2491	10.06	8.27						

**Table 9:** Performance comparison among octree implementing methods using Data 3.

	Memory-Based Octree			File-Based Octree			Semi-Isometric Octree Group $\left(t_{i}=2\right)$		
**Depth**	Memory usage (MB)	Construction time (s)	Proximity operation time (s)	Memory usage (MB)	Construction time (s)	Proximity operation time (s)	Memory usage. (MB)	Construction time (s)	Proximity operation time (s)
8	8718	44.63	0.30	2584	46.82	36.47	3370	58.70	3.46
9	8890	51.47	0.14	2756	53.57	11.79	5299	76.17	3.67
10	9392	60.61	0.19	3258	62.68	7.24			
11	10962	74.01	0.61	4828	75.96	7.07			
12	15048	94.72	2.64						
13	22289	131.93	13.68						

**Table 10:** Performance comparison among isometric octree groups using Data 1.

	$t_{i}=1$			$t_{i}=2$			$t_{i}=3$		
**Depth**	Memory usage (MB)	Construction time (s)	Proximity operation time (s)	Memory usage (MB)	Construction time (s)	Proximity operation time (s)	Memory usage. (MB)	Construction time (s)	Proximity operation time (s)
8	3324	59.80	4.18	3025	58.41	9.47	2913	57.97	12.87
9	4272	69.92	2.70	3477	66.80	4.24	3242	65.47	7.29
10	6912	84.83	1.98	4784	78.56	2.45	4115	76.35	2.82
11				8240	99.67	2.14	6515	95.63	2.36

**Table 11:** Performance comparison among isometric octree groups using Data 2.

	$t_{i}=1$			$t_{i}=2$			$t_{i}=3$		
**Depth**	Memory usage (MB)	Construction time (s)	Proximity operation time (s)	Memory usage (MB)	Construction time (s)	Proximity operation time (s)	Memory usage. (MB)	Construction time (s)	Proximity operation time (s)
8	211	3.84	2.59	187	3.68	5.54	183	3.65	7.64
9	301	4.60	2.08	223	4.29	2.65	205	4.18	4.07
10	598	5.65	2.25	364	5.15	2.20	292	4.91	2.60
11							574	5.94	2.62

**Table 12:** Performance comparison among isometric octree groups using Data 3.

	$t_{i}=1$			$t_{i}=2$			$t_{i}=3$		
**Depth**	Memory usage (MB)	Construction time (s)	Proximity operation time (s)	Memory usage (MB)	Construction time (s)	Proximity operation time (s)	Memory usage. (MB)	Construction time (s)	Proximity operation time (s)
8	4893	65.04	3.67	3370	58.70	3.46	3007	56.41	4.48
9				5299	76.17	3.67	4101	70.67	3.53
10							7289	91.43	4.15
11									
